# Three-Dimensional MoS_2_/Reduced Graphene Oxide Nanosheets/Graphene Quantum Dots Hybrids for High-Performance Room-Temperature NO_2_ Gas Sensors

**DOI:** 10.3390/nano12060901

**Published:** 2022-03-09

**Authors:** Cheng Yang, Yanyan Wang, Zhekun Wu, Zhanbo Zhang, Nantao Hu, Changsi Peng

**Affiliations:** 1 School of Optoelectronic Science and Engineering & Collaborative Innovation Center of Suzhou Nano Science and Technology, Soochow University, Suzhou 215006, China; 20205239020@stu.suda.edu.cn (C.Y.); 20185208036@stu.suda.edu.cn (Z.W.); 20205239028@stu.suda.edu.cn (Z.Z.); changsipeng@suda.edu.cn (C.P.); 2 Key Lab of Advanced Optical Manufacturing Technologies of Jiangsu Province & Key Lab of Modern Optical Technologies of Education Ministry of China, Soochow University, Suzhou 215006, China; 3 Key Laboratory of Thin Film and Microfabrication (Ministry of Education), Department of Micro/Nano Electronics, School of Electronic Information and Electrical Engineering, Shanghai Jiao Tong University, Shanghai 200240, China

**Keywords:** three-dimensional structure, MoS_2_, reduced graphene oxide (rGO), graphene quantum dots (GQDs), gas sensor

## Abstract

This study presents three-dimensional (3D) MoS_2_/reduced graphene oxide (rGO)/graphene quantum dots (GQDs) hybrids with improved gas sensing performance for NO_2_ sensors. GQDs were introduced to prevent the agglomeration of nanosheets during mixing of rGO and MoS_2_. The resultant MoS_2_/rGO/GQDs hybrids exhibit a well-defined 3D nanostructure, with a firm connection among components. The prepared MoS_2_/rGO/GQDs-based sensor exhibits a response of 23.2% toward 50 ppm NO_2_ at room temperature. Furthermore, when exposed to NO_2_ gas with a concentration as low as 5 ppm, the prepared sensor retains a response of 15.2%. Compared with the MoS_2_/rGO nanocomposites, the addition of GQDs improves the sensitivity to 21.1% and 23.2% when the sensor is exposed to 30 and 50 ppm NO_2_ gas, respectively. Additionally, the MoS_2_/rGO/GQDs-based sensor exhibits outstanding repeatability and gas selectivity. When exposed to certain typical interference gases, the MoS_2_/rGO/GQDs-based sensor has over 10 times higher sensitivity toward NO_2_ than the other gases. This study indicates that MoS_2_/rGO/GQDs hybrids are potential candidates for the development of NO_2_ sensors with excellent gas sensitivity.

## 1. Introduction

Nowadays, with industrialization and the continuous development of technology and science, the detection of nitrogen oxides (NO_X_) has attracted increasing attention [1,2,3]. Due to environmental and health concerns, the development of a high-sensitivity gas sensor that can accurately, reliably, and quickly detect low-concentration NO_2_ gas is essential for air quality monitoring and protection of human health [4,5].

Up to now, a variety of materials have been used to synthesize NO_2_ sensors, including metal oxides [6], conducting polymers [7], nanocarbon materials [8], and transitional metal dichalcogenides [9]. Among these, graphene has received widespread attention as a potential gas-sensing material. Being a typical *p*-type semiconductor material, reduced graphene oxide (rGO) exhibits more structural defects and dangling bonds than pure GO, which offers advantageous conditions for gas adsorption [10,11,12]. However, pure rGO-based sensors generally show poor gas sensitivity toward NO_2_ gas at room temperature [13]. Therefore, several researchers have tried to combine rGO with other nanomaterials to improve its gas-sensing performance [14,15,16]. It has been shown that the three-dimensional (3D) nanostructure of rGO composite can accelerate electron transport and improve the conductivity of composite materials.

In recent years, few-layer or single-layer two-dimensional transition metal sulfides, including TiS_2_ [17], WS_2_ [18], MoSe_2_ [19], MoS_2_ [20], and WSe_2_ [21], have attracted increasing attention from the academic community. Among these, MoS_2_ has been widely used as a gas-sensitive material in various gas monitoring applications because of its low cost, unique electronic structure, and suitable bandgap. However, the pure MoS_2_ material exhibits few accessible active sites, poor conductivity, and restacking of aggregations, which hinder electron transport and gas adsorption [22,23]. Introduction of a second component to form binary hybrids is considered an effective route to tackle these issues. Many low-dimensional nanomaterials, including graphene [24], carbon nanotubes [25], carbon dots [26], graphene quantum dots [27], etc., have been used to improve the gas sensing performance of MoS_2_ nanosheets through the formation of hybrid structures [28,29,30].

GQDs, with a size smaller than 20 nm, possess numerous characteristics in common with graphene, including their boundary effects and unique quantum confinement effects. Hence, they are widely used in biology, materials, chemistry, and other fields [31,32,33]. Several studies have shown that small-size graphene has high conductivity and superior electron transport ability, which contribute to its gas sensitivity [34,35]. Binary hybrids of MoS_2_ with rGO and GQDs have been proposed to improve the conductivity, increase the number of active sites, and accelerate electron transport. The addition of rGO nanosheets and GQDs can effectively avoid the agglomeration of MoS_2_, thereby supplying many binding sites for the adsorption of gas molecules.

In this study, alternately stacked 3D structures based on MoS_2_/rGO/GQDs ternary hybrids are prepared for NO_2_ gas sensing. The introduction of GQDs can prevent the agglomeration of MoS_2_ and rGO nanosheets. The rGO nanosheets serve as a channel for carrier transmission and a substrate for the growth of MoS_2_ nanoflowers. Additionally, the 3D nanostructure of the composite material provides numerous good adsorption sites for NO_2_ gas. These sites are beneficial for electron transmission and further enhance the gas-sensing properties of the MoS_2_/rGO/GQDs hybrids. The results reveal that the MoS_2_/rGO/GQDs-based sensor has high-magnitude response, good selectivity, excellent stability, and quick response toward NO_2_ at room temperature.

## 2. Materials and Methods

### 2.1. Chemical Reagents

Sodium molybdate dihydrate (H_4_MoNa_2_O_6_), thiourea (CH_4_N_2_S), hydrochloric acid (HCl), ethanol (C_2_H_6_O), sodium hydroxide (NaOH), and polyvinylpyrrolidone (PVP) were obtained from Sinopharm Chemical Reagent Co., Ltd. (Shanghai, China). Benzopyrene was purchased from Tokyo Chemical Industry (Tokyo, Japan). Nitric acid (HNO_3_, 65–68%) was obtained from Chinasun Specialty Products Co., Ltd. (Changshu, China). None of the above chemical reagents required further purification.

### 2.2. Fabrication of the GQDs

The GQDs were synthesized using a modified version of the method reported by Wang [36]. Typically, 1 g of benzopyrene was added to 80 mL of HNO_3_ and stirred for 12 h at 80 °C to be nitrated. The mixed solution was washed by deionized water several times, repeatedly filtered using 0.22 µm microfiltration membranes until the filtrate became colorless, and freeze-dried at −52 °C for 12 h. Then, 1.5 g of the resulting yellowish powder was dispersed into 300 mL of NaOH solution (2 mol/L) via ultrasonication for 3 h and stirred for 5 h. The resulting liquid was poured into a reactor lined with polytetrafluoroethylene and kept in an oven for 8 h at 200 °C. After cooling down to room temperature, the obtained solution was filtered through a 0.22 μm microporous membrane with deionized water. In order to remove the unfused small molecules and sodium salts, the filtrate was subjected to a dialysis treatment for two days in a dialysis bag, and it was freeze-dried to obtain a dark brown GQDs powder. Finally, the obtained GQDs powder was dispersed into deionized water to form a GQDs solution of 1 mg/mL for later use.

### 2.3. Fabrication of the MoS_2_ Nanoflowers

The MoS_2_ nanoflowers were prepared via the hydrothermal process. As in a typical procedure, 1.21 g of sodium molybdate dihydrate and 2.36 g of thiourea were weighed into a beaker and prepared into a 40 mL solution. After 3 h stirring, the mixture was poured into an autoclave lined with polytetrafluoroethylene and placed in an oven for 12 h at 200 °C. When the solution cooled down to room temperature, the yellow supernatant was removed with a dropper. The bottom sediment was washed several times with ethanol and deionized water and collected by centrifugation. The resultant powder was transferred to a vacuum drying oven and dried for 8 h at 60 °C to obtain black MoS_2_ powder.

### 2.4. Fabrication of the MoS_2_/rGO Nanocomposites

GO was synthesized using an improved version of Hummers’ method [37]. To synthesize MoS_2_/rGO nanocomposites, 75 mg of MoS_2_ powder was dispersed into 25 mL of deionized water and continuously stirred for 1 h. Then, 15 mL of about 1 mg/mL GO solution was added. After 8 h of stirring, the mixture was placed in a reaction kettle and kept in an oven for 12 h at 200 °C. Subsequently, after cooling down to room temperature, the supernatant liquid was removed by a dropper. The product was then centrifuged at 10,000 rpm for 5 min and washed three times with deionized water. After repeated centrifugation and washing cycles, the black precipitate was placed in a vacuum drying oven and then dried for 8 h at 80 °C to obtain MoS_2_/rGO nanocomposites.

### 2.5. Fabrication of the MoS_2_/rGO/GQDs Hybrids

The MoS_2_ powder, GO, and GQDs dispersion obtained through the above steps were used to prepare the MoS_2_/rGO/GQDs hybrids. Briefly, 15 mL of the GQDs dispersion (1 mg/mL) was dispersed into the obtained MoS_2_/rGO nanocomposites. The suspension was then sonicated for 3 h and stirred for 5 h to achieve a homogeneous solution. The obtained solution was placed into a polytetrafluoroethylene-lined reactor and heated for 12 h at 200 °C. Subsequently, the reactor was cooled down to room temperature. The supernatant liquid was removed using a straw, and the remaining solution was centrifuged for 10 min at 10,000 rpm. The precipitate was washed several times with ethanol and deionized water and separated by centrifugation. Then, it was dispersed into an appropriate amount of deionized water with a concentration of 1 mg/mL. The resulting thick solution was placed in a refrigerator to be frozen and then freeze-dried for 16 h to prepare the foamy MoS_2_/rGO/GQDs hybrids. The obtained MoS_2_, rGO, and GQDs were admixed at a mass ratio of 50:10:10 at 200 °C for 12 h in the hydrothermal process. The obtained MoS_2_/rGO/GQDs hybrids are here referred to as MoS_2_/rGO/GQDs-1. For comparison, hybrids with mass ratios of 50:5:5, 50:3:3, and 50:2:2 were also synthesized and labeled as MoS_2_/rGO/GQDs-2, MoS_2_/rGO/GQDs-3, and MoS_2_/rGO/GQDs-4, respectively.

### 2.6. Characterization

The morphology and structure were examined via atomic force microscopy (AFM, Dimension Icon, Bruker, Billerica, MA, USA) and scanning electron microscopy (SEM, Sigma300, Carl Zeiss, Oberkochen, Germany). A laser microscope confocal Raman spectrometer (*λ* = 514 nm, HR800, HORIBA Jobin Yvon, France) was used to acquire the Raman spectra. An X-ray diffractometer (XRD, XPert-Pro MPD, Panalytical, Holland) was used to analyze the crystal structures. The atomic valence and molecular structure of the samples were determined via X-ray photoelectron spectroscopy (XPS, ESCALAB250XI, Thermo Fisher Scientific, Waltham, MA, USA).

### 2.7. Fabrication and Measurement of the Synthesized Sensors

**Figure 1:**
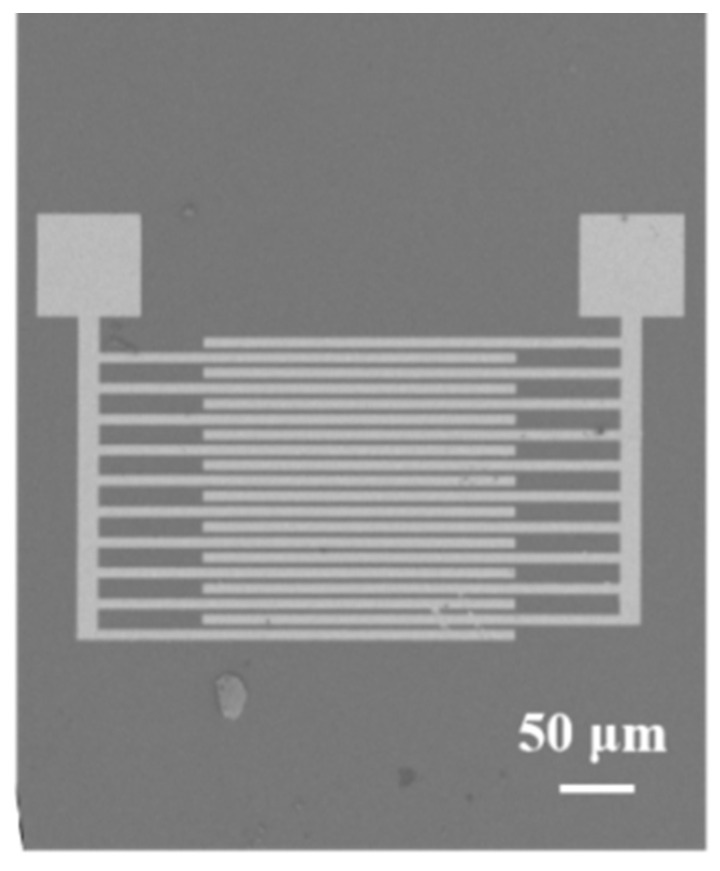
SEM image of the interdigitated electrode.

The MoS_2_/rGO and MoS_2_/rGO/GQDs hybrids were used as the gas-sensitive materials for the synthesis of NO_2_ sensors. Traditional microfabrication procedures were adopted. First, the silicon wafer was hydrophilized, dried, and spin-coated with photoresist. It was then exposed and developed with a mask. After Au sputtering and degumming, interdigitated electrode fingers were obtained. Figure 1 shows that the resultant electrode is about 720 μm long and 600 μm wide. An appropriate amount of the previously synthesized MoS_2_/rGO/GQDs solution was dropped onto the interdigitated electrode using a microsyringe and dried for the subsequent NO_2_ gas-sensitivity test.

The gas-sensitivity measurement was carried out through a high-precision semiconductor tester (Agilent 4156C, Santa Clara, CA 95051, USA). During the measurement, the voltage was set to 5 mV, and the current change was recorded by the sensing device in real time. NO_2_ gas was used as the test gas, and different concentrations of NO_2_ gas were obtained by regulating the flow ratio between the background gas and NO_2_ gas. At the beginning of the test, the background gas was introduced for 100 s to maintain the output current in a stable range, and then, the background gas together with NO_2_ gas were introduced at a certain proportion. *R*_0_ refers to the initial resistance value of the measured sensor under the background gas, while *R* denotes the real-time resistance value of the measured sensor exposed to NO_2_ gas. The sensitivity can then be defined as *S* = (*R* − *R_0_*)/*R_0_* × 100%.

## 3. Results and Discussion

### 3.1. Nanocomposite Material Characterization

**Figure 2:**
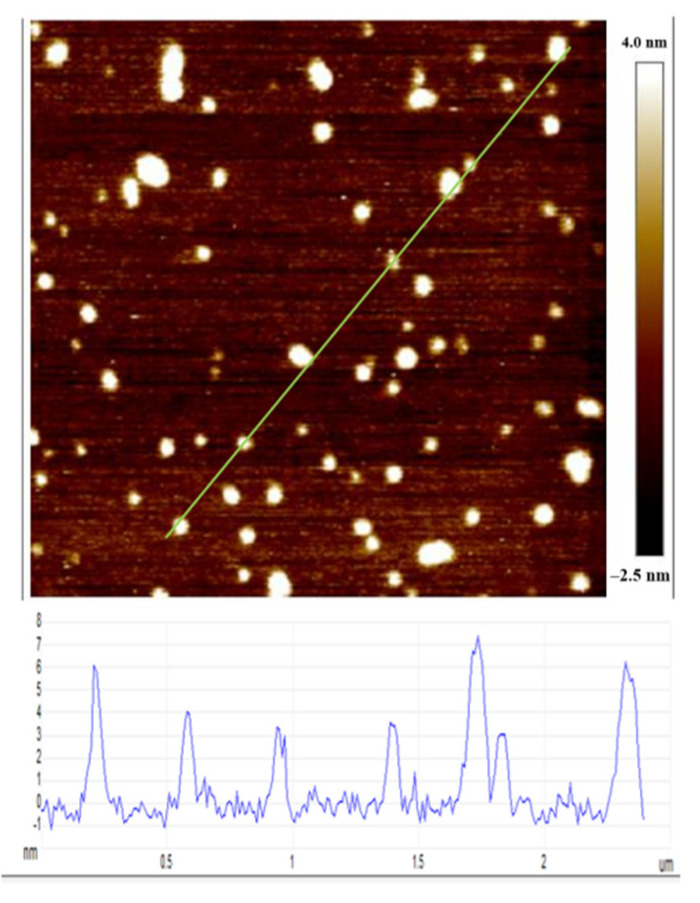
AFM image of GQDs.

AFM measurements were conducted to characterize the morphology of the prepared GQDs. The GQDs were more similar to discs rather than spherical objects. Figure 2 shows that the diameters of the GQDs were in a range from 2 to 7 nm, and the average diameter was about 4 nm. Furthermore, the aggregation of the GQDs can also be observed in Figure 2. This aggregation may be due to the weak hydrogen bonds or the noncovalent bond interactions among the oxygenated functional groups that are present in the GQDs [38,39]. The AFM morphology measurement of MoS_2_/rGO/GQDs hybrids was also performed and an image is provided in the supplementary information (Figure S1) to confirm the existence of GQDs in the MoS_2_/rGO/GQDs hybrids.

**Figure 3:**
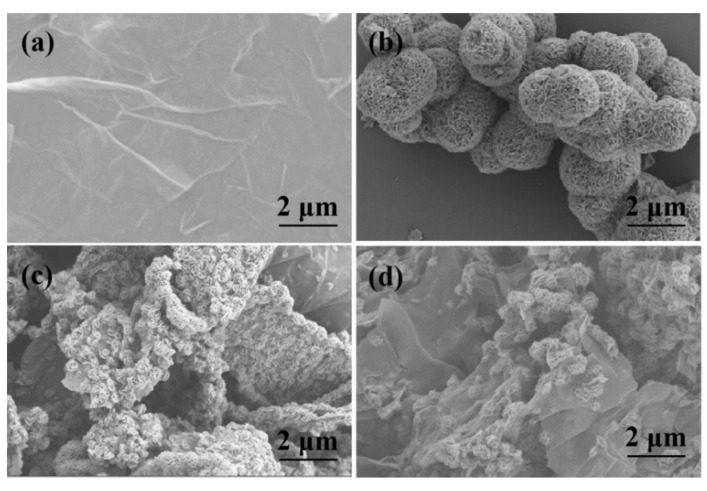
SEM images of (**a**) GO, (**b**) MoS_2_, (**c**) MoS_2_/rGO and (**d**) MoS_2_/rGO/GQDs.

The microstructure of the prepared composites was characterized through SEM. GO exhibits nearly transparent nanosheets with many wrinkles, as shown in Figure 3a [40,41]. It can be seen from Figure 3b that the pure MoS_2_ nanoflowers formed by the layered nanosheets have noticeable ripples, and their diameter is about 500 nm. It can be clearly observed from Figure 3c that the MoS_2_ nanoflowers anchored on the surface of rGO nanosheets are not uniformly distributed. Instead, they are stacked together and aggregated into nanospheres with the rGO nanosheets. The morphology of the MoS_2_/rGO/GQDs hybrids is shown in Figure 3d. It can be observed that the introduction of GQDs greatly improves the homogeneous distribution of rGO and MoS_2_ nanosheets. Additionally, numerous 3D interconnected foldable nanostructures are present in the MoS_2_/rGO/GQDs hybrids. The MoS_2_ nanoflowers and the small GQDs particles in the MoS_2_/rGO/GQDs hybrids are distributed on the exposed active sites of the rGO nanosheets. The introduction of GQDs provides nucleation sites for the formation of MoS_2_/rGO nanocomposites and prevents their agglomeration [42]. The morphologies of MoS_2_/rGO/GQDs hybrids with different ratios of GQDs are given in the supporting information (Figure S2).

**Figure 4:**
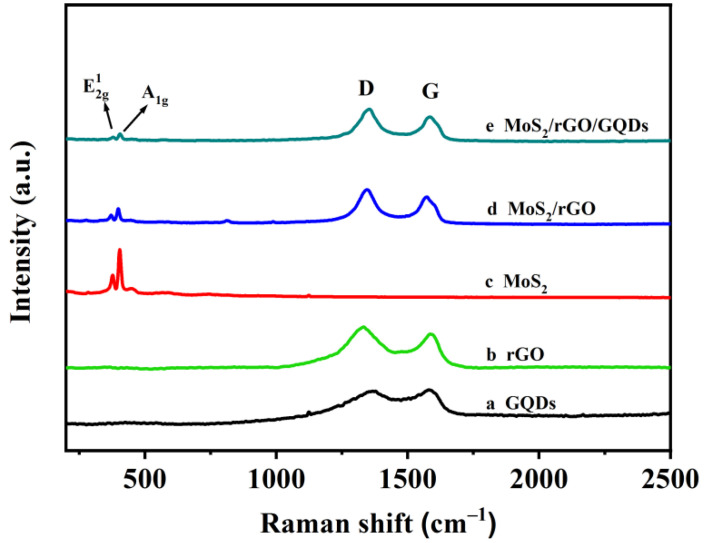
Raman spectra of (**a**) GQDs, (**b**) rGO, (**c**) MoS_2_, (**d**) MoS_2_/rGO, and (**e**) MoS_2_/rGO/GQDs.

As shown in Figure 4, Raman spectroscopy was used to detect the nonpolar vibrations between the same type of atom in the samples. The Raman spectra of GQDs, rGO, MoS_2_/rGO, and MoS_2_/rGO/GQDs show two characteristic peaks at ~1350 and ~1580 cm^−1^ corresponding to the D and G bands of graphene, respectively. The D-band can be attributed to the disorder degree or edge folding degree of graphene, whereas the G-band is due to the first-order scattering of the E_2g_ mode. Usually, the intensity ratio of the D-band and G-band (*I*_D_/*I*_G_) reveals the extent of graphene reduction [43,44]. As shown in Figure 4, the *I*_D_/*I*_G_ values of GQDs, rGO, MoS_2_/rGO, and MoS_2_/rGO/GQDs are 0.975, 1.226, 1.244, and 1.035, respectively. In comparison with MoS_2_/rGO, it can be clearly observed that the *I*_D_/*I*_G_ value of MoS_2_/rGO/GQDs decreased from 1.244 to 1.035, implying that some of the defects of rGO were removed during the deposition of GQDs. The decrease in *I_D_*/*I_G_* ratio proves that GQDs are successfully fabricated onto the MoS_2_/rGO/GQDs hybrids [45,46]. The peaks of pure MoS_2_ are located at 377 and 403 cm^−1^, which correspond to the $E_{2g}^{1}$ and A_1g_ vibrational modes, respectively [47]. The $E_{2g}^{1}$ peak corresponds to the Mo–S in-plane vibration of the MoS_2_ lattice, while the A_1g_ peak is attributed to the Mo–S out-of-plane vibration [48]. In contrast to pure MoS_2_, the values of the $E_{2g}^{1}$ and A_1g_ peaks in the MoS_2_/rGO and MoS_2_/rGO/GQDs samples are significantly reduced, which confirms that GQDs effectively inhibit the aggregation of MoS_2_.

**Figure 5:**
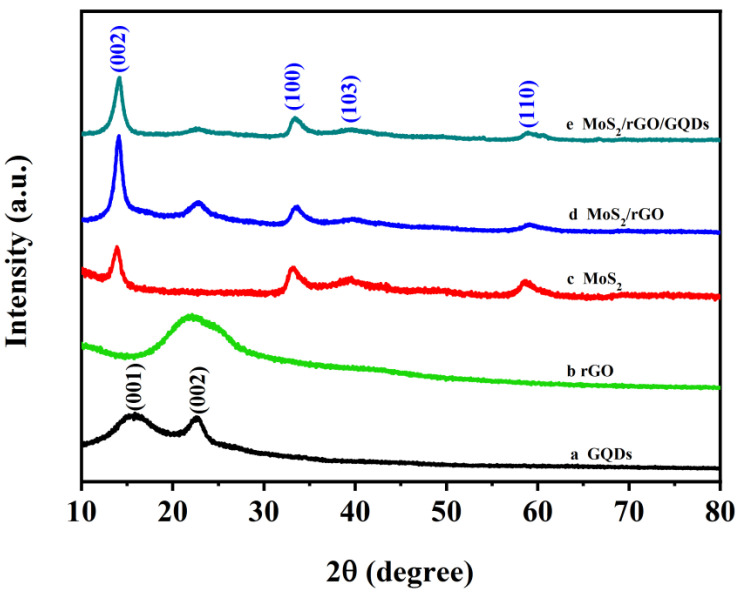
XRD patterns of (**a**) GQDs, (**b**) rGO, (**c**) MoS_2_, (**d**) MoS_2_/rGO, and (**e**) MoS_2_/rGO/GQDs.

The crystallinity and crystal phase of the hybrids were revealed via XRD. Figure 5a shows that the GQDs have two broad diffraction peaks at 15.8° and 22.6°, which correspond to the (001) and (002) crystal planes, respectively [49]. As displayed in Figure 5b, the four diffraction peaks at 13.9°, 33.3°, 39.6° and 58.8° correspond to the (002), (100), (103) and (110) planes, which are in agreement with the standard JCPDS card of 2H–MoS_2_ (JCPDS No.37-1492) [50]. All the peaks of pure MoS_2_ are also observed in the XRD patterns of the MoS_2_/rGO and MoS_2_/rGO/GQDs samples, which indicates the successful formation of the MoS_2_ nanoflowers in these samples. In comparison with the sharp diffraction peak of pure bulk MoS_2_ located at 2θ = 13.9°, the diffraction peaks of MoS_2_/rGO and MoS_2_/rGO/GQDs are wider, which may be due to the poorer crystallinity of the obtained samples and the decrease in particle size [51]. Furthermore, a diffraction peak was observed around 22.5° in rGO, MoS_2_/rGO and MoS_2_/rGO/GQDs samples corresponding to the (002) plane of rGO, which confirms the presence of rGO and the successful reduction of GO in the composite material [52]. It can also be seen that the diffraction peak of MoS_2_/rGO nanocomposites located at 2θ = 22.5° shifted slightly to 2θ = 22.9° when GQDs were added to the MoS_2_/rGO nanocomposites. This shift was due to the higher functionality of GQDs, as the surface groups cause an increase in the lattice parameter of the rGO nanosheets [53]. This result indicates the successful incorporation of GQDs into the MoS_2_/rGO nanocomposites. It is also important to note that compared to MoS_2_/rGO nanocomposites, the peak intensity decreases for MoS_2_/rGO/GQDs hybrids, which verifies that the addition of GQDs can effectively avoid the agglomeration of MoS_2_ nanoflowers and rGO nanosheets. The XRD results demonstrate that the MoS_2_/rGO/GQDs hybrids were successfully fabricated.

**Figure 6:**
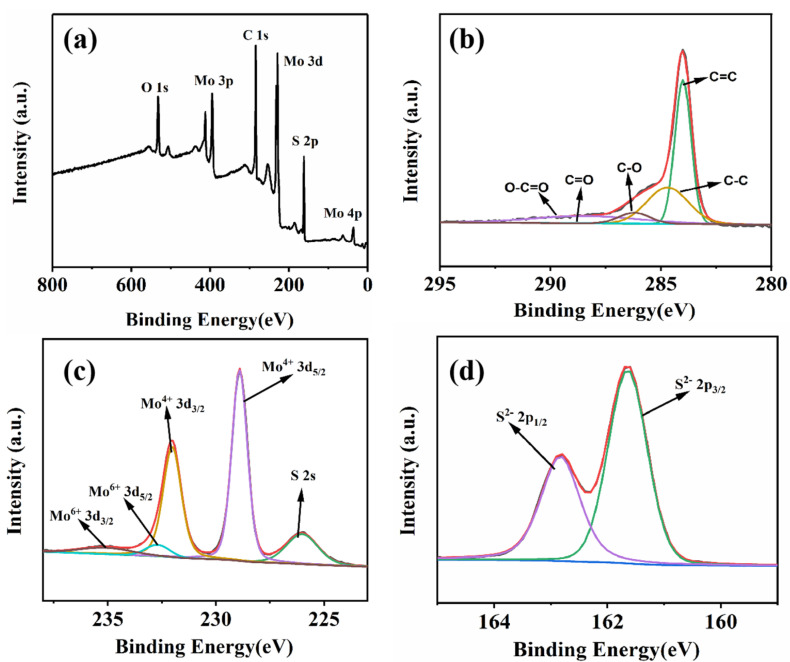
XPS spectrum of (**a**) MoS_2_/rGO/GQDs, (**b**) C 1s XPS spectrum of MoS_2_/rGO/GQDs, (**c**) Mo 3d XPS spectrum of MoS_2_/rGO/GQDs and (**d**) S 2p XPS spectrum of MoS_2_/rGO/GQDs.

The MoS_2_/rGO/GQDs hybrids were analyzed via XPS. Figure 6a shows the spectrum of the MoS_2_/rGO/GQDs hybrids. It can be clearly seen that the MoS_2_/rGO/GQDs hybrids contain oxygen, carbon, sulfur, and molybdenum elements. Figure 6b illustrates that the C 1s energy spectrum of the MoS_2_/rGO/GQDs hybrids can also be deconvolved into five peaks: C=C (284.6 eV), C–C (285.2 eV), C–O (286.8 eV), C=O (288.4 eV), and O–C=O (289.3 eV). In Figure 6c, two characteristic orbital peaks can be observed at 232.0 eV (3d_3/2_) and 228.9 eV (3d_5/2_), which are ascribed to the Mo^4+^ ions of MoS_2_ [54]. Additionally, the two small Mo^6+^ peaks at 235.1 eV (3d_3/2_) and 232.7 eV (3d_5/2_) confirm the presence of Mo–O bonds, which may be caused by residual MoO_4_^2−^ in the precursor [55]. The presence of S 2s in MoS_2_ is confirmed by the small peak located at 226 eV [56]. In Figure 6d, the two dominant S 2p_1/2_ and S 2p_3/2_ peaks at 162.8 eV and 161.6 eV are attributed to the divalent sulfide ions (S^2−^) in MoS_2_ [57]. These XPS results prove the successful synthesis of the MoS_2_/rGO/GQDs hybrids using the hydrothermal process.

**Figure 7:**
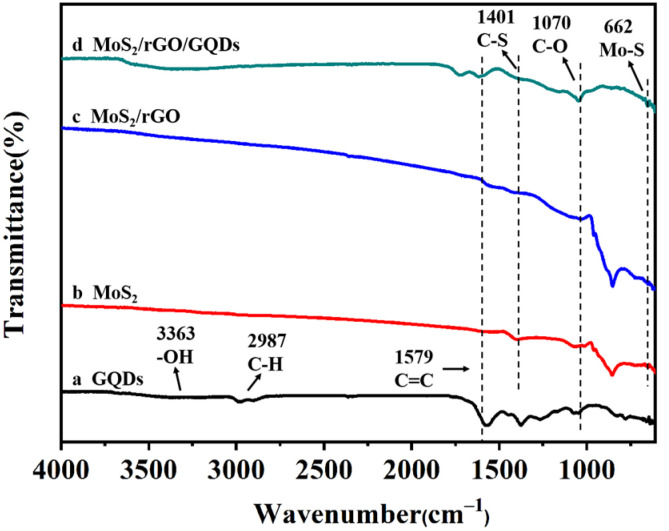
FT-IR spectra of GQDs MoS_2_, MoS_2_/GQDs and MoS_2_/rGO/GQDs.

Figure 7 shows the FTIR spectra of GQDs, MoS_2_, MoS_2_/rGO, and MoS_2_/rGO/GQDs. The sharp peak of the GQDs at 1579 cm^−1^ is related to the C=C stretching vibration [58], suggesting that the GQDs are mainly composed of C=C bonds. The peaks located at 1070, 2987, and 3363 cm^−1^ are related to the C–O, C–H, and –OH stretching vibrations [59,60], respectively. The peaks corresponding to the C–S and Mo–S bonds of MoS_2_ are located at 1401 and 662 cm^−1^. The C=C stretching vibration of the GQDs can also be observed at 1579 cm^−1^ in the MoS_2_/rGO/GQD hybrids, which indicates the existence of GQDs in the composite materials. The common peaks at 1401 and 662 cm^−1^ in the MoS_2_/rGO and MoS_2_/rGO/GQD hybrids are related to the C–S and Mo–S bonds, which confirms the presence of MoS_2_ [61]. It is noteworthy that the characteristic peaks associated with oxygenated groups, such as O–H, C=O, and C–O groups, were not clearly observed in the MoS_2_/rGO nanocomposites, which verifies that GO was successfully reduced via the hydrothermal approach [62].

### 3.2. Gas-Sensing Properties

**Figure 8:**
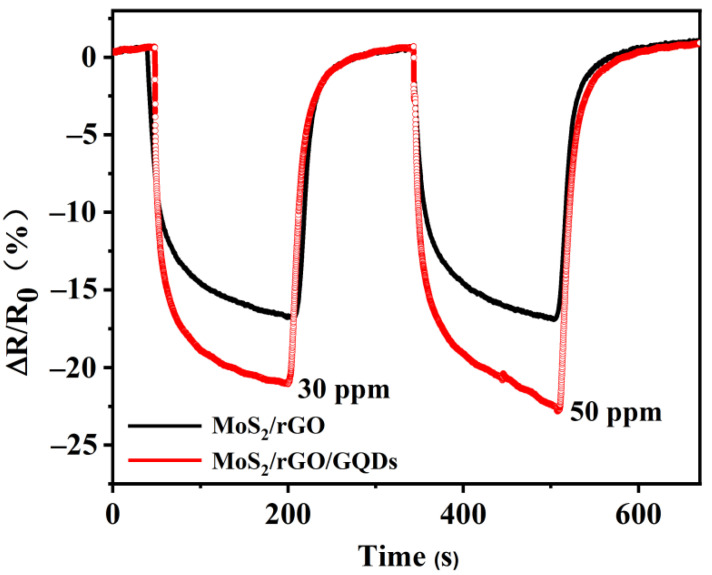
Response and recovery curves of MoS_2_/rGO and MoS_2_/rGO/GQD-based sensors exposed to 30 and 50 ppm NO_2_.

First, the gas-sensing performance was tested by detecting NO_2_ gas at room temperature. The experiment results show that pure GQD materials have basically no response to NO_2_ gas. This may be due to the smaller size of the GQDs compared with that of the interdigitated electrode, which makes it difficult to form a stable conductive loop. The response values of the MoS_2_/rGO and MoS_2_/rGO/GQDs hybrids exposed to 30 and 50 ppm NO_2_ gas at room temperature are shown in Figure 8. The MoS_2_/rGO nanocomposites showed 16.8% and 16.9% response values to 30 and 50 ppm NO_2_. Compared with the MoS_2_/rGO nanocomposites, the addition of GQDs improved sensitivity to 21.1% and 23.2% when the sensor was exposed to 30 and 50 ppm NO_2_ gas, respectively. The GQDs also act as active sites, which can prevent the agglomeration of nanosheets during mixing of rGO and MoS_2_ and provide numerous reaction sites for NO_2_ gas adsorption. Consequently, the gas-sensing performance of the hybrids is enhanced [63].

Generally, materials with a large effective surface area can provide more active parts for gas adsorption and interaction, which contributes to the enhanced gas sensing performance. A CV test was carried out to confirm the effective surface area of hybrids with and without GQDs. Values of 2.253 and 1.165 can be calculated from the Randles–Sevcik equation (see details in the supporting information, Figures S3 and S4), indicating a great enhancement of effective surface area was achieved after the addition of GQDs [64,65,66].

**Figure 9:**
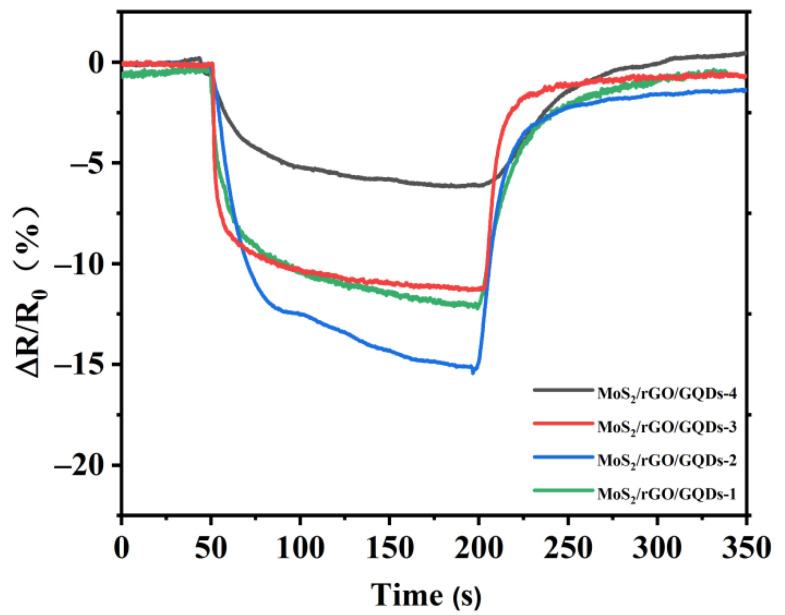
Response and recovery curves of MoS_2_/rGO/GQDs-based sensors exposed to 5 ppm NO_2_.

As is well known, the nanostructure of gas-sensitive materials is crucial to improving the gas-sensing properties of gas sensors [67,68]. The MoS_2_/rGO/GQDs hybrids with 3D nanostructures were expected to exhibit higher gas sensitivity. In order to explore the effect of the GQDs content on the gas sensitivity, four types of MoS_2_/rGO/GQDs hybrids were synthesized by changing the mass ratio of MoS_2_, rGO, and GQDs from 50:10:10 to 50:2:2. These prepared composites are sequentially labeled MoS_2_/rGO/GQDs-1, MoS_2_/rGO/GQDs-2, MoS_2_/rGO/GQDs-3, and MoS_2_/rGO/GQDs-4. Figure 9 shows the comparison between the responses of these different gas sensors. The response values toward 5 ppm NO_2_ within 150 s were 12.1%, 15.2%, 11.3%, and 6.2% for MoS_2_/rGO/GQDs-1, MoS_2_/rGO/GQDs-2, MoS_2_/rGO/GQDs-3, and MoS_2_/rGO/GQDs-4, respectively. The resistance of all samples decreased remarkably when the sensor was exposed to NO_2_ gas and rapidly returned to the initial value after stopping the exposure. The MoS_2_/rGO/GQDs-2-based sensor exhibited better gas sensitivity than the MoS_2_/rGO/GQDs-1-based sensor, which indicates that the uniform distribution of MoS_2_ nanoflowers in the hybrids can effectively improve the gas-sensing performance of the composites. The enhanced gas sensitivity can be attributed to the interaction of the MoS_2_ nanoflowers with the rGO nanosheets, which results in the construction of a 3D network and improves the interconnectivity among MoS_2_, rGO, and GQDs [69]. However, with a further decrease in the content of GQDs in the hybrids, the gas sensitivity of the MoS_2_/rGO/GQDs-3 and MoS_2_/rGO/GQDs-4 hybrids decreased to 11.3% and 6.2%, respectively. Thus, the aggregation and restacking of MoS_2_ nanoflowers are not conducive to improving gas adsorption. A possible reason for this behavior may be that the aggregation of MoS_2_ nanoflowers weakens the supporting effect of the rGO nanosheets and reduces the probability of NO_2_ gas adsorption on the heterogeneous interface between the MoS_2_ nanoflowers and the rGO nanosheets, thereby causing a decrease in gas sensitivity [70]. Therefore, due to its highest response value, MoS_2_/rGO/GQDs-2 was selected to further study the gas-sensing performance.

For the purpose of comparison, the response curves towards 5 ppm NO_2_ of the pristine MoS_2_/rGO with various mass ratios of MoS_2_ and rGO are shown in the supplementary information (Figure S5). The response values to 5 ppm NO_2_ gas at room temperature were 7.2%, 10.4%, 9.5%, and 5.2% for the MoS_2_/rGO-based sensors with the mass ratio of MoS_2_ and rGO varying from 50:10 to 50:2, respectively. The response values of pristine MoS_2_/rGO samples were lower than those of MoS_2_/rGO/GQDs hybrids, indicating that the gas-sensing properties of the MoS_2_/rGO/GQDs-based sensors can be effectively facilitated by the addition of GQDs.

**Figure 10:**
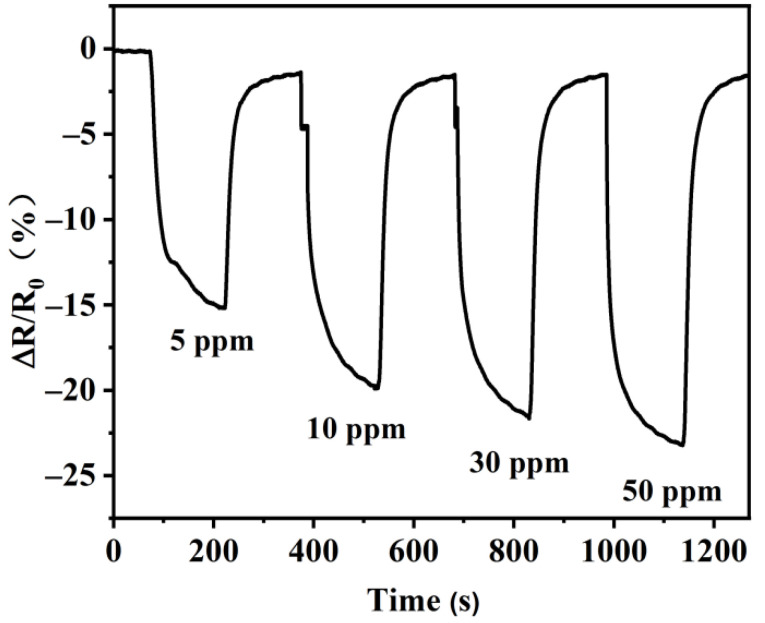
Response and recovery curves of the MoS_2_/rGO/GQDs-2 sensor towards different concentrations of NO_2_.

Figure 10 displays the dynamic gas-sensitive response curve of the sensor based on the MoS_2_/rGO/GQDs-2 hybrids to different NO_2_ gas concentrations at room temperature. As mentioned in previous reports, the gas sensitivity at high NO_2_ gas concentrations is stronger than that at low concentrations because of adsorption and desorption during the gas sensitivity tests [71,72]. At NO_2_ concentrations of 50, 30, 10, and 5 ppm, the response values were 23.2%, 21.1%, 19.9%, and 15.2%, respectively. The response values dropped with the decrease in NO_2_ gas concentration. The response and recovery times remained stable at 150 s. It can be clearly observed that the gas-sensitive response of the MoS_2_/rGO/GQDs-based sensor decreased sharply when exposed to NO_2_ and recovered immediately after the exposure was stopped. Compared to the previous results, the MoS_2_/rGO/GQDs-based sensor exhibited lower operating temperature, higher response, and lower detection limit (Table 1).

**Figure 11:**
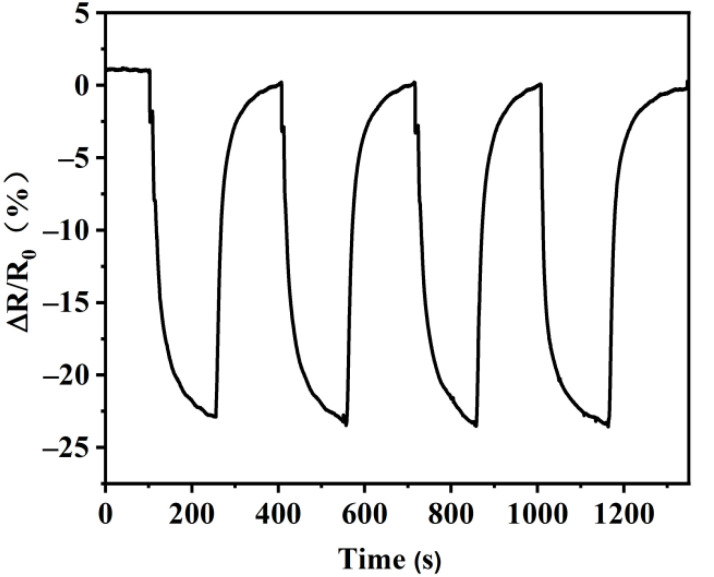
Reproducibility of response after exposure to 50 ppm NO_2_.

To test the stability and repeatability of the MoS_2_/rGO/GQDs-2-based sensor response, its gas-sensing properties were measured in four consecutive dynamic response processes. Figure 11 displays the stability and repeatability of the response of the MoS_2_/rGO/GQDs-based sensor exposed to 50 ppm NO_2_ gas at room temperature. The gas sensitivity, response time, and recovery time of the sensor did not change significantly after four cycles. After three cycles, the gas-sensitive response retained a value of 23.2%, which indicates the excellent repeatability and stability of the MoS_2_/rGO/GQDs hybrids.

**Figure 12:**
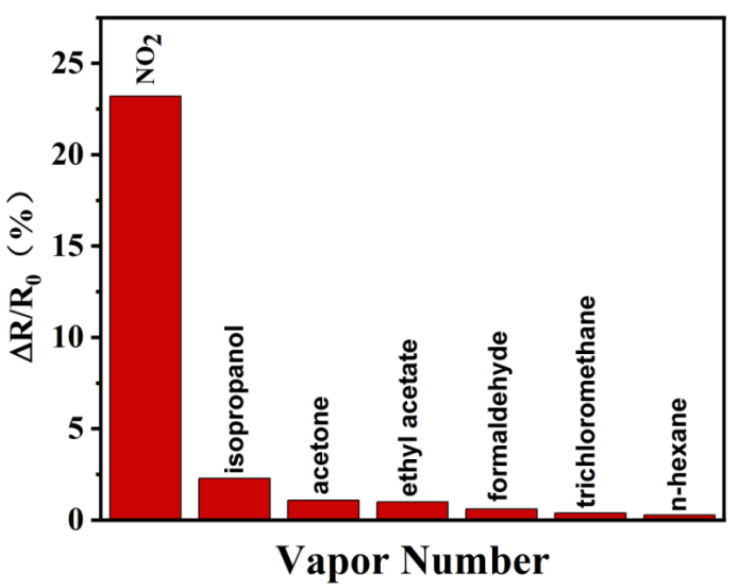
Selectivity toward different kinds of gases.

In practical applications, NO_2_ is not found alone but is often accompanied by many other toxic and harmful gases. Thus, selectivity is another important indicator typically used to evaluate the performance of gas sensors in practical applications [73]. To explore the gas selectivity of the obtained MoS_2_/rGO/GQDs hybrids, the sensor was used to measure several conventional industrial organic gases, including isopropanol, acetone, formaldehyde, ethyl acetate, trichloromethane, and n-hexane. The saturated vapor in the solvent bottle was diluted with N_2_ to a concentration of 1%. Even though the concentration of these vapors was greater than that of NO_2_, the test results revealed that the response value to 50 ppm NO_2_ gas was more than 10 times the response to other vapors, as displayed in Figure 12. It can be concluded that the MoS_2_/rGO/GQDs hybrids have an outstanding reactivity to NO_2_ gas, while the reactivity to other vapors is negligible. Therefore, the experimental results demonstrate that the MoS_2_/rGO/GQDs-based sensor exhibits high selectivity toward NO_2_ and can be used in practical applications.

### 3.3. Gas-Sensing Mechanism

**Figure 13:**
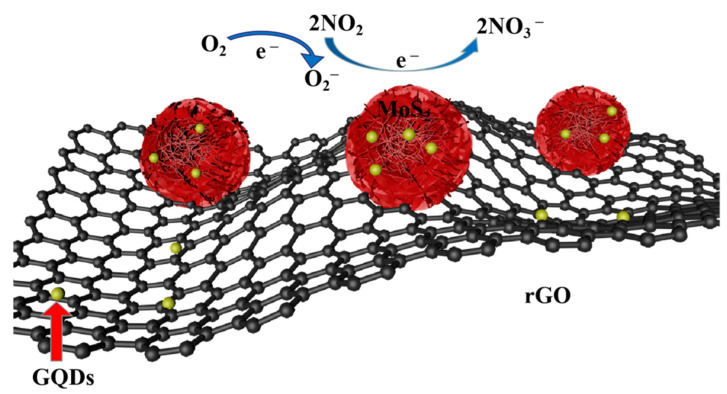
Schematic illustration of the gas sensing mechanism for MoS_2_/rGO/GQDs composites.

It can be seen from Figure 13 that the generation of the heterojunction and the modification of the GQDs are responsible for the improvement in gas-sensing properties. The ternary combination of MoS_2_, GQDs, and rGO results in the formation of many nanostructures similar to *p*–*n* junctions at the interfaces. These special nanostructures are essential to improve the electron transmission efficiency. When the MoS_2_/rGO/GQDs hybrids were exposed to background gas, oxygen was adsorbed onto the surface of the hybrids because of the strong adsorption properties of rGO and MoS_2_. Since rGO has a greater work function value (*W* *=* 4.7 eV) than MoS_2_ (*W* = 4.3 eV), electrons are transferred from the conduction band of MoS_2_ to the conduction band of rGO until the Fermi level equilibrium is reached [74,75]. In the meantime, the oxygen molecules attached to the surface of the MoS_2_/rGO/GQDs hybrids are converted into oxygen anions ($O_{2}^{-}$) after capturing electrons, owing to their high affinity toward electrons. The reaction is the following:

(1) $$O_{2}+e^{-}\to O_{2}^{-}$$

The generation of oxygen anions ($O_{2}^{-}$) reduces the concentration of electrons; this is the reason why the resistance of the fabricated MoS_2_/rGO/GQDs hybrids was relatively high in the background gas. When the MoS_2_/rGO/GQDs hybrids were exposed to NO_2_, the NO_2_ molecules underwent a reaction with the minority carriers (electrons) and the oxygen ions ($O_{2}^{-}$) [76] adsorbed onto the MoS_2_/rGO/GQDs hybrids to generate ${\mathrm{NO}}_{3}^{-}$ ions as follows:

(2) $$2{\mathrm{NO}}_{2}+O_{2}^{-}+e^{-}\to 2{\mathrm{NO}}_{3}^{-}$$

Due to this reaction, the captured electrons travel back to the conduction band of MoS_2_ to increase the electron concentration in the hybrids. The above reaction leads to decreased thickness of the charge layer between MoS_2_ and rGO, thereby decreasing the resistance of the obtained sensor. In addition, the GQDs modification on the surface of the MoS_2_/rGO heterojunction is also responsible for improving the sensing performance. The GQDs serve as an electron mediator at the interface heterojunction, and supply numerous active sites for the hybrids. The active sites allow chemisorbed oxygen to react with NO_2_, which further improves the gas sensitivity of the hybrids [77,78].

## 4. Conclusions

A novel 3D structured sensor based on MoS_2_/rGO/GQDs hybrids was prepared for detecting NO_2_ at room temperature. The MoS_2_/rGO/GQDs hybrids were obtained by anchoring MoS_2_ nanoflowers and GQDs nanoparticles onto rGO nanosheets. The introduction of the GQDs inhibited the agglomeration of the MoS_2_/rGO nanocomposites, considerably improved the homogeneous distribution of rGO and MoS_2_ nanosheets, and provided numerous reaction sites for NO_2_ gas adsorption. The prepared MoS_2_/rGO/GQDs-based sensor had a response of 23.2% toward 50 ppm NO_2_ gas, while it retained a response of 15.2% when exposed to NO_2_ concentration as low as 5 ppm. Furthermore, it was also found that the MoS_2_/rGO/GQDs-based sensor exhibited a very low detection limit, high response, excellent stability, outstanding repeatability, excellent selectivity, and quick response/recovery characteristics toward NO_2_ gas at room temperature. The superior gas-sensing ability was due to the synergistic effects of the 3D nanostructures, heterojunctions, and GQDs in the MoS_2_/rGO/GQDs hybrids. The proposed MoS_2_/rGO/GQDs-based sensor exhibits outstanding gas-sensing properties and, thus, has great potential in the detection of NO_2_ gas.

## Figures and Tables

**Table 1:** Comparison of main properties and performance characteristics of nanomaterials used to detect NO_2_ gas.

Material	Operating Temperature (°C)	Concentration	Sensitivity	Reference
NiO/SnO_2_/rGO	RT	60 ppm	62.27	[10]
MoS_2_/rGO	60 °C	2 ppm	59.8%	[22]
3D MoS_2_/rGO	80 °C	1 ppm	2483%	[54]
MoS_2_/WS_2_	RT	50 ppm	26.12	[56]
SnO_2_/(0.3%)rGO	RT	10 ppm	2.021	[68]
SnO_2_/rGO	200 °C	4 ppm	4.56	[70]
MoS_2_/rGO/GQDs	RT	50 ppm	23.2%	this work
MoS_2_/rGO/GQDs	RT	5 ppm	15.2%	this work

## Data Availability

Not applicable.

## Supplementary Materials

The following supporting information can be downloaded at: https://www.mdpi.com/article/10.3390/nano12060901/s1, Figure S1: AFM image of MoS_2_/rGO/GQDs; Figure S2: SEM images of (a) MoS_2_/rGO/GQDs-1, (b) MoS_2_/rGO/GQDs-2 (c) MoS_2_/rGO/GQDs-3 and (d) MoS_2_/rGO/GQDs-4; Figure S3: Cyclic voltammograms of (a) MoS_2_/rGO nanocomposites and (b) MoS_2_/rGO/GQDs hybrids in 10 mM [Fe(CN)6]^3−/4−^ and 0.1 M KCl solution at different scan rates from 25 to 300 mV·s^−1^; Figure S4: Peak currents as a function of scan rate for the determination of the effective surface area; Figure S5: Response and recovery curves of MoS_2_/rGO-based sensors to 5 ppm NO_2_.

## References

[1] Duy L.T., Kim D.J., Trung T.Q., Dang V.Q., Kim B.Y., Moon H.K., Lee N.E. (2014). High performance three-dimensional chemical sensor platform using reduced graphene oxide formed on high aspect-ratio micro-pillars. Adv. Funct. Mater..

[2] Liu B.L., Chen L., Liu G., Abbas A.N., Fathi M., Zhou C.W. (2014). High-performance chemical sensing using schottky-contacted chemical vapor deposition grown monolayer MoS_2_ transistors. ACS Nano.

[3] Casals O., Markiewicz N., Fabrega C., Gràcia I., Cané C., Wasisto H.S., Waag A., Prades J.D. (2019). A parts per billion (ppb) sensor for NO_2_ with microwatt (μW) power requirements based on micro light plates. ACS Sens..

[4] Xian F., Zong B., Mao S. (2018). Metal-organic framework-based sensors for environmental contaminant sensing. Nano-Micro Lett..

[5] Lustig W.P., Mukherjee S., Rudd N.D., Desai A.V., Li J., Ghosh S.K. (2017). Metal—Organic frameworks: Functional luminescent and photonic materials for sensing applications. Chem. Soc. Rev..

[6] Wang Z.H., Men G.L., Zhang R.X., Gu F.B., Han D.M. (2018). Pd loading induced excellent NO_2_ gas sensing of 3DOM In_2_O_3_ at room temperature. Sens. Actuators B Chem..

[7] Liu C.H., Tai H.L., Zhang P., Ye Z.B., Su Y.J., Jiang Y.D. (2017). Enhanced ammonia-sensing properties of PANI-TiO_2_-Au ternary self assembly nanocomposite thin film at room temperature. Sens. Actuators B Chem..

[8] Lee S.W., Lee W., Hong Y., Lee G., Yoon D.S. (2018). Recent advances in carbon material-based NO_2_ gas sensors. Sens. Actuators B Chem..

[9] Zhang D.Z., Sun Y.E., Jiang C.X., Yao Y., Wang D.Y., Zhang Y. (2017). Room-temperature highly sensitive CO gas sensor based on Ag-loaded zinc oxide/molybdenum disulfifide ternary nanocomposite and its sensing properties. Sens. Actuators B Chem..

[10] Zhang J., Wu J.J., Wang X.X., Zeng D.W., Xie C.S. (2017). Enhancing room-temperature NO_2_ sensing properties via forming heterojunction for NiO-rGO composited with SnO_2_ nanoplates. Sens. Actuators B Chem..

[11] Yin F.F., Li Y., Yue W.J., Gao S., Zhang C.W., Chen X.Z. (2020). Sn_3_O_4_/rGO heterostructure as a material for formaldehyde gas sensor with a wide detecting range and low operating temperature. Sens. Actuators B Chem..

[12] Han J.T., Kim B.J., Kim B.G., Kim J.S., Jeong B.H., Jeong S.Y., Jeong H.J., Cho J.H., Lee G.W. (2011). Enhanced electrical properties of reduced graphene oxide multilayer films by in-situ insertion of a TiO_2_ layer. ACS Nano.

[13] Chen Z., Wang J., Pan D., Wang Y., Noetzel R., Li H., Xie P., Pei W.L., Umar A., Jiang L. (2018). Mimicking a dog’s nose: Scrolling graphene nanosheets. ACS Nano.

[14] Zhao B., Xu Y.T., Huang S.Y., Zhang K., Yuen M.M.F., Xu J.B., Fu X.Z., Sun R., Wong C.P. (2016). 3D RGO frameworks wrapped hollow spherical SnO_2_-Fe_2_O_3_ mesoporous nano-shells: Fabrication, characterization and lithium storage properties. Electrochim. Acta.

[15] Wang Z.Y., Gao S., Fei T., Liu S., Zhang T. (2019). Construction of ZnO/SnO_2_ heterostructure on reduced graphene oxide for enhanced nitrogen dioxide sensitive performances at room temperature. ACS Sens..

[16] Mao S., Cui S.M., Lu G.H., Yu K.H., Wen Z.H., Chen J.H. (2012). Tuning gas sensing properties of reduced graphene oxide using tin oxide nanocrystals. J. Mater. Chem..

[17] Das T., Chakraborty S., Ahuja R., Kawazoe Y., Das G.P. (2020). Charge transfer driven interaction of CH_4_, CO_2_ and NH_3_ with TiS_2_ monolayer: Influence of vacancy defect. Catal. Today.

[18] Cao J., Wang W., Zhou J., Chen J., Deng H., Zhang Y., Liu X. (2020). Controllable gas sensitive performance of 1T’ WS_2_ monolayer instructed by strain: First-principles simulations. Chem. Phys. Lett..

[19] Hu X.Y., Gui Y.G., Liu Y.J., Ran L., Chen X.P. (2021). Adsorption characteristics of H_2_S, SO_2_, SO_2_F_2_, SOF_2_, and N_2_ on NiO–MoSe_2_ monolayer for gas-sensing applications. Vacuum.

[20] Zhao P.C., Ni M.J., Xu Y.T., Wang C.X., Chen C., Zhang X.R., Li C.Y., Xie Y.X., Fei J.J. (2019). A novel ultrasensitive electrochemical quercetin sensor based on MoS_2_ carbon nanotube@graphene oxide nanoribbons/HS-cyclodextrin/graphene quantum dots composite fifilm. Sens. Actuators B Chem..

[21] Lu Z., Zhai Y., Liang Q.Z., Wu W. (2020). Promoting sensitivity and selectivity of NO_2_ gas sensor based on metal (Pt, Re, Ta)-doped monolayer WSe_2_: A DFT study. Chem. Phys. Lett..

[22] Zhou Y., Liu G.Q., Zhu X.Y., Guo Y.C. (2017). Ultrasensitive NO_2_ gas sensing based on rGO/MoS_2_ nanocomposite fifilm at low temperature. Sens. Actuators B Chem..

[23] Chen C.Z., Shen M., Li Y.Z. (2021). One pot synthesis of 1T@2H-MoS_2_/SnS_2_ heterojunction as a photocatalyst with excellent visible light response due to multiphase synergistic effect. Chem. Phys..

[24] Geng X., Lu P.F., Zhang C., Lahem D., Olivier M.G., Debliquy M. (2019). Room-temperature NO_2_ gas sensors based on rGO@ZnO_1−x_ composites: Experiments and molecular dynamics simulation. Sens. Actuators B Chem..

[25] Drozdowska K., Rehman A., Krajewska A., Lioubtchenko D.V., Paviov K., Rumyantsev S., Smulko J., Cywainski G. (2021). Effects of UV light irradiation on fluctuation enhanced gas sensing by carbon nanotube networks. Sens. Actuators B Chem..

[26] Supchocksoonthorn P., Hanchaina R., Sinoy M.C.A., Luna M.D.G.D., Kangamasksin T., Paoprasert P. (2021). Novel solution- and paper-based sensors based on label-free fluorescent carbon dots for the selective detection of pyrimethanil. Appl. Surf. Sci..

[27] Ibrahim I., Lim H.N., Huang N.M. (2020). In-situ formation of electron acceptor to inhibit charge separation of photo-electrochemical sensor of dopamine-based CdS/Au/GQDs. Electrochim. Acta.

[28] Lu S., Chen M.Z., Wang Y.L., Li R., Lin J., Zhang X.T. (2021). Highly efficient MoS_2_/rGO electrocatalysts for triiodide reduction as Pt-free counter electrode for dye-sensitized solar cells. Sol. Energy.

[29] Lv K.L., Suo W.Q., Shao M.D., Zhu Y., Wang X.P., Feng J.J., Fang M.W. (2019). Nitrogen doped MoS_2_ and nitrogen doped carbon dots composite catalyst forelectroreduction CO_2_ to CO with high Faradaic efficiency. Nano Energy.

[30] Wu H., Guo Z.S., Li M., Hu G.H., Tang T., Wen J.F., Li X.Y., Huang H.F. (2021). Enhanced pseudocapacitive performance of MoS_2_ by introduction of both N-GQDs and HCNT for flexible supercapacitors. Electrochim. Acta.

[31] Wei L.S., Cai J.H., Li X.Y., Wang X.Y. (2019). Fabrication of graphene quantum dots/chitosan composite film and its catalytic reduction for 4-nitrophenol. Ferroelectrics.

[32] Shen J.H., Zhu Y.H., Yang X.L., Li C.Z. (2012). Graphene quantum dots: Emergent nanolights for bioimaging, sensors, catalysis and photovoltaic devices. Chem. Commun..

[33] Ponomarenko L.A., Schedin F., Katsnelson M.I., Yang R., Hill E.W., Novoselov K.S., Geim A.K. (2008). Chaotic dirac billiard in graphene quantum dots. Science.

[34] Wongrat E., Nuengnit T., Panyathip R., Chanlek N., Hongsith N., Choopun S. (2020). Highly selective room temperature ammonia sensors based on ZnO nanostructures decorated with graphene quantum dots (GQDs). Sens. Actuators B Chem..

[35] Chen Z.L., Wang D., Wang X.Y., Yang J.H. (2020). Preparation and formaldehyde sensitive properties of N-GQDs/SnO_2_ nanocomposite. Chin. Chem. Lett..

[36] Wang L., Wang Y.L., Xu T., Liao H.B., Yao C.J., Liu Y., Li Z., Chen Z.W., Pan D.Y., Sun L.T. (2014). Gram-scale synthesis of single-crystalline graphene quantum dots with superior optical properties. Nat. Commun..

[37] Bhangare B., Ramgir N.S., Pathak A., Sinju K.R., Debnath A.K., Jagtap S., Suzuki N., Muthe K.P., Terashima C., Aswal D.K. (2020). Role of sensitizers in imparting the selective response of SnO_2_/RGO based nanohybrids towards H_2_S, NO_2_ and H_2_. Mater Sci. Semicond. Process..

[38] Li L.L., Ji J., Fei R., Wang C.Z., Lu Q., Zhang J.R., Jiang L.P., Zhu J.J. (2012). A facile microwave avenue to electrochemiluminescent two-color graphene quantum dots. Adv. Funct. Mater..

[39] Chua C.K., Sofer Z., Simek P., Jankovsky O., Klimova K., Bakardjieva S., Kuckova S.H., Pumera M. (2015). Synthesis of strongly fluorescent graphene quantum dots by cage-opening buckminsterfullerene. ACS Nano.

[40] Zhang D.Z., Liu A.M., Chang H.Y., Xia B.K. (2015). Room-temperature high-performance acetone gas sensor based on hydrothermal synthesized SnO_2_ reduced graphene oxide hybrid composite. RSC Adv..

[41] Zhang D.Z., Yin N.L., Xia B.K. (2015). Facile fabrication of ZnO nanocrystalline-modifified graphene hybrid nanocomposite toward methane gas sensing application. J. Mater. Sci. Mater. Electron..

[42] Sun Q.H., Wu Z.F., Duan H.M., Jia D.Z. (2019). Detection of triacetone triperoxide (TATP) precursors with an array of sensors based on MoS2/RGO composites. Sensors.

[43] Yu X.L., Tang J., Terabe K., Sasaki T., Gao R.S., Ito Y., Nakura K., Asano K., Suzuki M. (2020). Fabrication of graphene/MoS_2_ alternately stacked structure for enhanced lithium storage. Mater. Chem. Phys..

[44] Ren H.B., Gu C.P., Joo S.W., Cui J.Y., Sun Y.F., Huang J.R. (2018). Preparation of SnO_2_ nanorods on reduced graphene oxide and sensing properties of as-grown nanocomposites towards hydrogen at low working temperature. Mater. Express.

[45] Lecaros R.L.G., Bismonte M.E., Doma B.T., Hung W.S., Hu C.C., Tsai H.A., Huang S.H., Lee K.R., Lai J.Y. (2020). Alcohol dehydration performance of pervaporation composite membranes with reduced graphene oxide and graphene quantum dots homostructured fifiller. Carbon.

[46] Chen Q., Hu Y., Hu C.G., Cheng H.H., Zhang Z.P., Shao H.B., Qu L.T. (2014). Graphene quantum dots–three-dimensional graphene composites for high-performance supercapacitors. Phys. Chem. Chem. Phys..

[47] Niu Y., Wang R.G., Jiao W.C., Ding G.M., Hao L.F., Yang F., He X.D. (2015). MoS_2_ graphene fifiber based gas sensing devices. Carbon.

[48] Ma L., Ye J.B., Chen W.X., Chen D.Y., Lee J.Y. (2014). Gemini surfactant assisted hydrothermal synthesis of nanotile-like MoS_2_/graphene hybrid with enhanced lithium storage performance. Nano Energy.

[49] Li Y., Hu Y., Zhao Y., Shi G.Q., Deng L., Hou Y.B., Qu L.T. (2011). An electrochemical avenue to green-luminescent graphene quantum dots as potential electron-acceptors for photovoltaics. Adv. Mater..

[50] Li W.Z., Li F., Wang X., Tang Y., Yang Y.Y., Gao W.B., Li R. (2017). A facile lyophilization synthesis of MoS_2_ QDs@graphene as a highly active electrocatalyst for hydrogen evolution reaction. Appl. Surf. Sci..

[51] Zhang K., Ye M.Q., Han A.J., Yang J.L. (2019). Preparation, characterization and microwave absorbing properties of MoS_2_ and MoS_2_-reduced graphene oxide (RGO) composites. J. Solid State Chem..

[52] Xu X.B., Sun Y., Qiao W., Zhang X., Chen X., Song X.Y., Wu L.Q., Zhong W., Du Y.W. (2017). 3D MoS_2_-graphene hybrid aerogels as catalyst for enhanced efficient hydrogen evolution. Appl. Surf. Sci..

[53] Riaz R., Ali M., Sahito I.A., Arbab A.A., Maiyalagan T., Anjum A.S., Ko M.J., Jeong S.H. (2019). Self-assembled nitrogen-doped graphene quantum dots (N-GQDs) over graphene sheets for superb electro-photocatalytic activity. Appl. Surf. Sci..

[54] Chen T.D., Yan W.H., Xu J.G., Li J.H., Zhang G.P., Ho D. (2019). Highly sensitive and selective NO_2_ sensor based on 3D MoS_2_/rGO composites prepared by a low temperature self-assembly method. J. Alloys Compd..

[55] Long L.N., Thi P.T., Kien P.T., Trung P.T., Ohta M., Kumabe Y. (2019). Controllable synthesis of MoS_2_/graphene lowdimensional nanocomposites and their electrical properties. Appl. Surf. Sci..

[56] Lkram M., Liu L.J., Liu Y., Ma L.F., Lv H., Ullah M., He L., Wu H.Y., Wang R.H., Shi K.Y. (2019). Fabrication and characterization of a high-surface area MoS_2_@WS_2_ heterojunction for the ultrasensitive NO_2_ detection at room temperature. J. Mater. Chem. A.

[57] Li W.R., Xu H.Y., Zhai T., Yu H.Q., Chen Z.R., Qiu Z.W., Song X.P., Wang J.Q., Cao B.Q. (2017). Enhanced triethylamine sensing properties by designing Au@SnO_2_/MoS_2_ nanostructure directly on alumina tubes. Sens. Actuators B Chem..

[58] Sun Y.Q., Wang S.Q., Li C., Luo P.H., Tao L., Wei Y., Shi G.Q. (2013). Large scale preparation of graphene quantum dots from graphite with tunable fluorescence properties. Phys. Chem. Chem. Phys..

[59] Shen J.H., Zhu Y.H., Yang X.L., Zong J., Zhang J.M., Li C.Z. (2012). One-pot hydrothermal synthesis of graphene quantum dots surface-passivated by polyethylene glycol and their photoelectric conversion under near-infrared light. New J. Chem..

[60] Xie M.M., Su Y.J., Lu X.N., Zhang Y.Z., Yang Z., Zhang Y.F. (2013). Blue and green photoluminescence graphene quantum dots synthesized from carbon fifibers. Mater. Lett..

[61] Hou X.H., Wang Z.W., Fan G.J., Ji H.P., Yi S.S., Li T., Wang Y., Zhang Z.T., Yuan L., Zhang R. (2020). Hierarchical three-dimensional MoS_2_/GO hybrid nanostructures for triethylamine-sensing applications with high sensitivity and selectivity. Sens. Actuators B Chem..

[62] Wang T., Sun Z., Huang D., Yang Z., Ji Q., Hu N.T., Yin G.L., He D.N., Wei H., Zhang Y.F. (2017). Studies on NH_3_ gas sensing by zinc oxide nanowire-reduced graphene oxide nanocomposites. Sens. Actuators B Chem. B.

[63] Wang C.X., Jin J.L., Sun Y.Y., Yao J.R., Zhao G.Z., Liu Y.Q. (2017). In-situ synthesis and ultrasound enhanced adsorption properties of MoS_2_/graphene quantum dot nanocomposite. Chem. Eng. J..

[64] Huang D., Yang Z., Li X.L., Zhang L.L., Hu J., Su Y.J., Hu N.T., Yin G.L., He D.N., Zhang Y.F. (2017). Three-dimensional conductive networks based on stacked SiO2@graphene frameworks for enhanced gas sensing. Nanoscale.

[65] Silamabarasan K., Harish S., Hara K., Archana J., Navaneethan M. (2021). Ultrathin layered MoS_2_ and N-doped graphene quantum dots (N-GQDs) anchored reduced graphene oxide (rGO) nanocomposite-based counter electrode for dye-sensitized solar cells. Carbon.

[66] Balamurugan J., Peera S.G., Guo M., Nguyen T.T., Kim N.H., Lee J.H. (2017). A hierarchical 2D Ni-Mo-S nanosheet@nitrogen doped graphene hybrid as a Pt-free cathode for high-performance dye sensitized solar cells and fuel cells. J. Mater. Chem. A.

[67] Wang Z.Y., Zhang T., Han T.Y., Fei T., Liu S., Lu G.Y. (2018). Oxygen vacancy engineering for enhanced sensing performances: A case of SnO_2_ nanoparticles-reduced graphene oxide hybrids for ultrasensitive ppb-level room-temperature NO_2_ sensing. Sens. Actuators B Chem..

[68] Gui Y.H., Wang H.Y., Tian K., Yang L.L., Guo H.S., Zhang H.Z., Fang S.M., Wang Y. (2018). Enhanced gas sensing properties to NO_2_ of SnO_2_/rGO nanocomposites synthesized by microwave-assisted gas-liquid interfacial method. Ceram. Int..

[69] Wan K.C., Yang J.L., Wang D., Wang X.Y. (2020). Graphene oxide@3D hierarchical SnO_2_ nanofifiber/nanosheets nanocomposites for highly sensitive and low-temperature formaldehyde detection. Molecules.

[70] Bhangare B., Ramgir N.S., Jagtap S., Debnath A.K., Muthe K.P., Terashima C., Aswal D.K., Gosavi S.W., Fujishima A. (2019). XPS and Kelvin probe studies of SnO_2_/RGO nanohybrids based NO_2_ sensors. Appl. Surf. Sci..

[71] Jiang L.L., Tu S.H., Yu H.T., Meng Y.M., Zhao Y.S., Hou X.G. (2020). Gas sensitivity of heterojunction TiO_2_NT/GO materials prepared by a simple method with low-concentration acetone. Ceram. Int..

[72] Wang S., Wang P., Li Z., Xiao C., Xiao B., Zhao R., Yang T., Zhang M. (2014). Facile fabrication and enhanced gas sensing properties of In_2_O_3_ nanoparticles. New J. Chem..

[73] Zhang S.S., Zhang B., Sun G., Li Y.W., Zhang B., Wang Y., Gao J.L., Zhang Z.Y. (2019). One-step synthesis of Ag/SnO_2_/rGO nanocomposites and their trimethylamine sensing properties. Mater. Res. Bull..

[74] Gong Y.X., Wang Y., Sun G., Jia T.K., Jia L., Zhang F.M., Lin L., Zhang B.Q., Cao J.L., Zhang Z.Y. (2018). Carbon nitride decorated ball-flflower like Co_3_O_4_ hybrid composite: Hydrothermal synthesis and ethanol gas-sensing application. Nanomaterials.

[75] Ye Z., Tai H., Guo R., Yuan Z., Liu C., Su Y., Chen Z., Jiang Y. (2017). Excellent ammonia sensing performance of gas sensor based on graphene/titanium dioxide hybrid with improved morphology. Appl. Surf. Sci..

[76] Cho B., Yoon J., Lim S.K., Kim A.R., Kim D.H., Park S.G., Kwon J.D., Lee Y.J., Lee K.H., Lee B.H. (2015). Chemical sensing of 2D graphene/MoS_2_ heterostructure device. ACS Appl. Mater. Interface.

[77] Guo T., Wang L.N., Sun S., Wang Y., Chen X.L., Zhang K.N., Zhang D.X., Xue Z.H., Zhou X.B. (2019). Layered MoS_2_@graphene functionalized with nitrogen-doped graphene quantum dots as an enhanced electrochemical hydrogen evolution catalyst. Chin. Chem. Lett..

[78] Sangeetha M., Madhan D. (2020). Ultra sensitive molybdenum disulfide (MoS_2_)/graphene based hybrid sensor for the detection of NO_2_ and formaldehyde gases by fiber optic clad modified method. Opt. Laser Technol..

