# Supplementary material for: Three-Dimensional MoS2/Reduced Graphene Oxide Nanosheets/Graphene Quantum Dots Hybrids for High-Performance Room-Temperature NO2 Gas Sensors
Source: Nanomaterials (Basel). 2022 Mar 9;12(6):901. doi: 10.3390/nano12060901 (PMC8954772; doi:10.3390/nano12060901)
Supplement: Supplementary file 1 [file nanomaterials-12-00901-s001.zip › nanomaterials-1588811-supplementary.pdf]

Supplementary Material

Three-Dimensional MoS_2/Reduced Graphene Oxide Nanosheets/Graphene Quantum Dots Hybrids for High-Performance Room-Temperature NO_2 Gas Sensors

Cheng Yang [1,2], Yanyan Wang [1,2,*], Zhekun Wu [1,2], Zhanbo Zhang [1,2], Nantao Hu [3,*] and Changsi Peng [1,2]

[1] School of Optoelectronic Science and Engineering & Collaborative Innovation Center of Suzhou Nano Science and Technology, Soochow University, Suzhou 215006, China; 20205239020@stu.suda.edu.cn (C.Y.); 20185208036@stu.suda.edu.cn (Z.W.); 20205239028@stu.suda.edu.cn (Z.Z.); changsipeng@suda.edu.cn (C.P.)

[2] Key Lab of Advanced Optical Manufacturing Technologies of Jiangsu Province & Key Lab of Modern Optical Technologies of Education Ministry of China, Soochow University, Suzhou 215006, China

[3] Key Laboratory of Thin Film and Microfabrication (Ministry of Education), Department of Micro/Nano Electronics, School of Electronic Information and Electrical Engineering, Shanghai Jiao Tong University, Shanghai 200240, China

* Correspondence: yywang@suda.edu.cn (Y.W.); hunantao@sjtu.edu.cn (N.H.)

The formation of MoS_2/rGO/GQDs hybrids was noticeable from the AFM image in Fig. S1, from which the spots corresponding to the GQDs were clearly observed on the exterior surface of the pristine MoS_2/rGO.

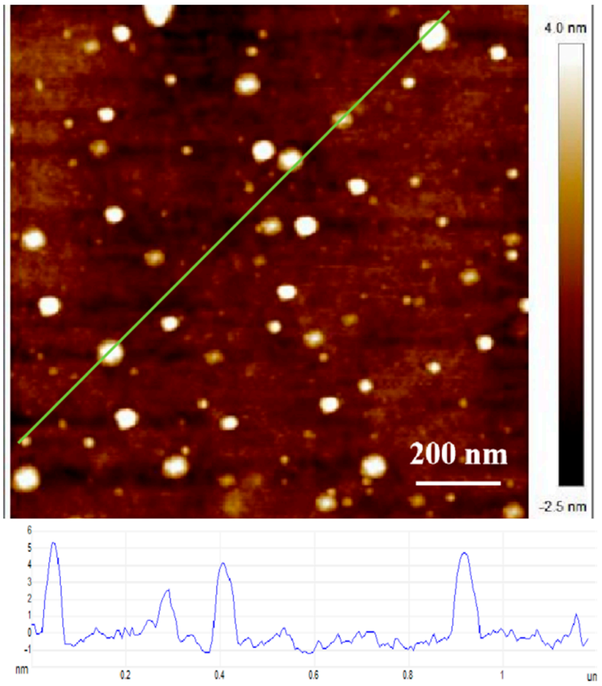

Figure S1. AFM image of MoS_2/rGO/GQDs.

Fig. S2 shows the morphologies of MoS_2/rGO/GQDs hybrids with different ratios of GQDs (MoS_2/rGO/GQDs hybrids with mass ratios of 50:10:10, 50:5:5, 50:3:3, and 50:2:2, denoted as MoS_2/rGO/GQDs-1, MoS_2/rGO/GQDs-2, MoS_2/rGO/GQDs-3, and MoS_2/rGO/GQDs-4, respectively). When the ratio of MoS_2, rGO and GQDs was 50:10:10, the dispersion of MoS_2/rGO/GQDs-1 hybrids was relatively uniform and MoS_2 nanoflowers combined closely with rGO nanosheets, without obvious agglomeration. When the ratio of MoS_2, rGO and GQDs was 50:5:5, numerous small MoS_2 nanoflowers were evenly distributed on the surface of rGO nanosheets in MoS_2/rGO/GQDs-2 hybrids. When the ratio of MoS_2, rGO and GQDs was 50:3:3, MoS_2 nanoflowers tended to aggregate in MoS_2/rGO/GQDs-3 hybrids. As the content of GQDs in the hybrids further decreased, MoS_2 nanosheets showed severe aggregation and overlapping. This caused an increase in the size of the formed MoS_2 nanoflowers and reduced the dispersion of the hybrids. The experimental results indicated that the microstructures of MoS_2/rGO/GQDs hybrids were significantly affected by the ratio of MoS_2, rGO and GQDs. As seen from Fig. S2, the addition of GQDs limited the aggregation of MoS_2 nanosheets, which caused a decrease in the size of MoS_2 nanoflowers. Numerous active sites and transportation channels were likely formed due to the small MoS_2 nanosheets, which contributed to the gas adsorption property. The MoS_2/rGO/GQDs-2 composite material exhibited better dispersion than the other hybrids and was selected for further study.

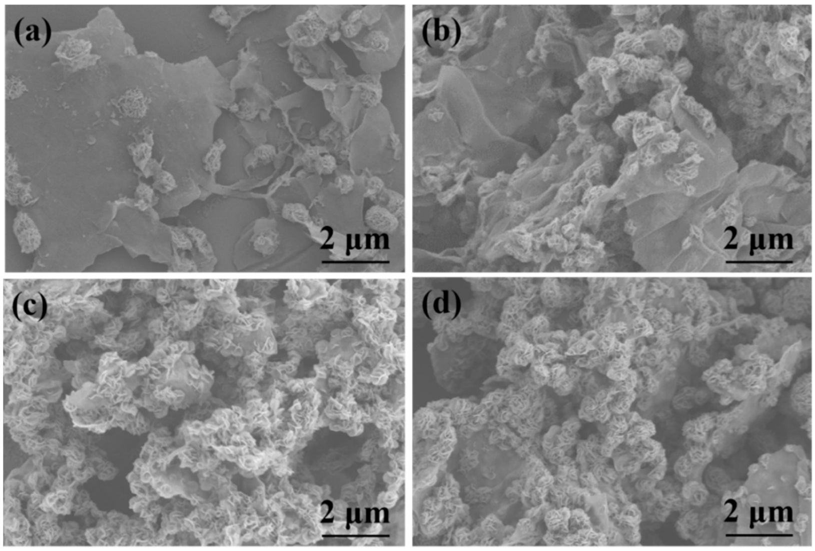

Figure S2. SEM images of (a) MoS_2/rGO/GQDs-1, (b) MoS_2/rGO/GQDs-2 (c) MoS_2/rGO/GQDs-3 and (d) MoS_2/rGO/GQDs-4.

CV tests were carried out in an aqueous solution of KCl and $K_3Fe(CN)_6$ to confirm whether the surface area increased after the addition of GQDs (Fig. S3). 200 μg of MoS_2/rGO nanocomposites and 200 μg of MoS_2/rGO/GQDs hybrids were uniformly coated on a 1 cm × 1 cm indium-tin-oxide (ITO) anode respectively for the CV test. The effective surface area can be calculated from the Randles-Sevcik equation [64]:

$$i_p = (2.687 \times 10^5) n^{3/2} v^{1/2} D^{1/2} AC$$

Where i_p refers to the peak current, n is the number of electrons transferred in the redox process ($n = 1$, in 10 mM $K_3Fe(CN)_6$), v is the scan rate, D is the diffusion coefficient ($D = 5.7 \times 10^{-6}$ cm^2s^{-1}, in 0.1 M KCl), A is the surface are and C is the concentration of $K_3Fe(CN)_6$. As shown in Fig. S4, the peak current i_p and the square root of scan rate v for both MoS_2/rGO/GQDs hybrids (slope = 14.455) and MoS_2/rGO nanocomposites (slope = 7.475). Moreover, the effective surface area of MoS_2/rGO/GQDs hybrids and MoS_2/rGO nanocomposites were calculated to be 2.253 and 1.165 cm^2, respectively, indicating the significant enhancement of effective surface area with the addition of GQDs [65,66].

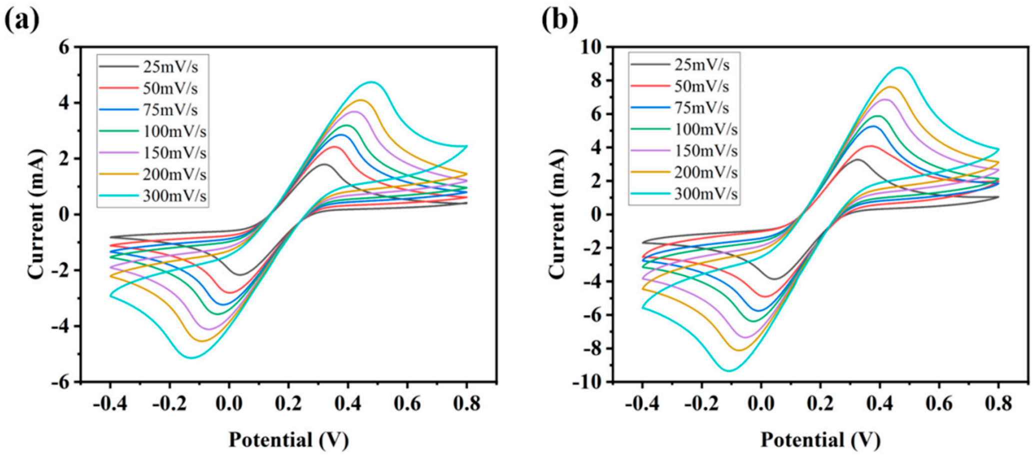

Figure S3. Cyclic voltammograms of (a) MoS_2/rGO nanocomposites and (b) MoS_2/rGO/GQDs hybrids in 10 mM $[Fe(CN)_6]^{3-/4-}$ and 0.1 M KCl solution at different scan rates from 25 to 300 $mV \cdot s^{-1}$.

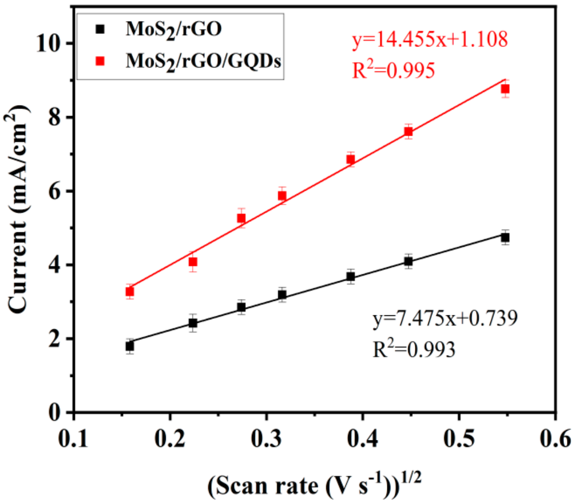

Figure S4. Peak currents as a function of scan rate for the determination of the effective surface area.

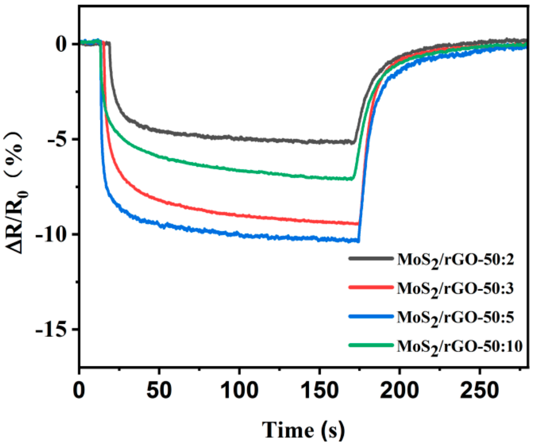

Figure S5. Response and recovery curves of MoS_2/rGO-based sensors to 5 ppm NO_2.
